# Selective Recovery of Cadmium, Cobalt, and Nickel from Spent Ni–Cd Batteries Using Adogen^®^ 464 and Mesoporous Silica Derivatives

**DOI:** 10.3390/ijms23158677

**Published:** 2022-08-04

**Authors:** Ahmed R. Weshahy, Ahmed K. Sakr, Ayman A. Gouda, Bahig M. Atia, H. H. Somaily, Mohamed Y. Hanfi, M. I. Sayyed, Ragaa El Sheikh, Enass M. El-Sheikh, Hend A. Radwan, Mohamed F. Cheira, Mohamed A. Gado

**Affiliations:** 1 Department of Chemistry, Faculty of Science, Zagazig University, Zagazig 44519, Egypt; 2 Department of Civil and Environmental Engineering, Wayne State University, 5050 Anthony Wayne Drive, Detroit, MI 48202, USA; 3 Nuclear Materials Authority, Cairo P.O. Box 530, Egypt; 4 Research Center for Advanced Materials Science (RCAMS), King Khalid University, P.O. Box 9004, Abha 61413, Saudi Arabia; 5 Department of Physics, Faculty of Science, King Khalid University, P.O. Box 9004, Abha 61413, Saudi Arabia; 6 Institute of Physics and Technology, Ural Federal University, St. Mira, 19, 620002 Yekaterinburg, Russia; 7 Department of Physics, Faculty of Science, Isra University, Amman 11622, Jordan

**Keywords:** spent Ni–Cd batteries, separation, cadmium, nickel, cobalt

## Abstract

Spent Ni–Cd batteries are now considered an important source for many valuable metals. The recovery of cadmium, cobalt, and nickel from spent Ni–Cd Batteries has been performed in this study. The optimum leaching process was achieved using 20% H_2_SO_4_, solid/liquid (S/L) 1/5 at 80 °C for 6 h. The leaching efficiency of Fe, Cd, and Co was nearly 100%, whereas the leaching efficiency of Ni was 95%. The recovery of the concerned elements was attained using successive different separation techniques. Cd(II) ions were extracted by a solvent, namely, Adogen^®^ 464, and precipitated as CdS with 0.5% Na_2_S solution at pH of 1.25 and room temperature. The extraction process corresponded to pseudo-2nd-order. The prepared PTU-MS silica was applied for adsorption of Co(II) ions from aqueous solution, while the desorption process was performed using 0.3 M H_2_SO_4_. Cobalt was precipitated at pH 9.0 as Co(OH)_2_ using NH_4_OH. The kinetic and thermodynamic parameters were also investigated. Nickel was directly precipitated at pH 8.25 using a 10% NaOH solution at ambient temperature. FTIR, SEM, and EDX confirm the structure of the products.

## 1. Introduction

Nowadays, cost-effective and low-cost techniques for collecting important metals from secondary sources have been developed to help meet economic and environmental constraints. In this regard, cadmium is prominent among non-ferrous metals. The principal application of cadmium has moved during the past 40 years from coatings to portable Ni–Cd batteries [1]. Rechargeable batteries (such as Ni–Cd batteries) have the highest quality, when utilized at low temperatures, which makes them a key user priority [2,3]. Thanks to their multiple attributes, i.e., long life spans, easy-of-rechargeing, no maintenance requirements, good shelf life, and reliability. The used Ni–Cd batteries wind up in large amounts in landfill, and cadmium waste pollutes the environment [4]. Spent Ni–Cd batteries are composed mostly of Ni–Cd electrode components corresponding to around 43–49% of battery weight. The remainder comprises the outer case steel parts/fundamental plates (40–49%) and feed- and Ni grids (9%), electrolyte, and 2% plastic constituents [5,6].

The recycling of spent Ni–Cd batteries has been thoroughly established and run commercially at scale [7,8,9,10,11]. Huge amounts of spent Ni–Cd batteries have been recycled internationally with pyrometallurgical procedures, including destructive cadmium distillation. The recycling process was established using demounting, crushing, metallurgic vacuum separation, and magnetic separation to produce environmentally friendly technology [12,13].

Acid leaching is a significant stage in hydrometallurgical improvements of the shredding of separated Ni–Cd battery powder. Cd and Ni efficiently dissolve during processes based on Ni–Cd wasted battery liquidation with hydrochloric acid [14,15,16]. The hydrochloric acid solution actually has a substantially higher leaching capacity than other acids; yet sulfuric acid has mostly been proposed as the total leaching and regeneration agent [17].

The separation and elimination of Cd from solutions involving various metal ions could be attained by adsorption, precipitation, ion exchange, electrolysis, solvent extraction, etc. [18,19]. With almost equivalent valence configurations, metal species allow co-transportation and selective extraction challenging. Selective extraction of Cd was possible in the presence of Ni, Mn, Zn, Fe, Mg, and Ca. However, Pb and Cu were co-extracted with Cd from processes established for HCl liquidation of Ni–Cd spent batteries [20]. Cd in the same mixture was observed to impair other metals’ physiological balance [21].

Solvent extraction is a very adaptable technique for the sometimes difficult extraction, purification, recovery, and separation of the aqueous medium incorporating metal ions [22]. It is a quick and straightforward, economical method [23]. Liquid-liquid extraction is employed for metal separation using immiscible fluids (usually one organic and another, an aqueous phase containing metal cations) in contact with each other. Most investigations employed the distribution ratios and metal separation factors with specified extractants for a particular metal [24,25]. Ionic liquids are task-specific extractants. They have useful and adaptable physicochemical properties, such as heat stability, high polarity, negligible steam pressure, inactivity, and a wide range of miscellaneous effects on other organic solvents, which have been highlighted in various scientific publications [26,27]. Adogen^®^ 464 has been widely investigated in the extraction of metal chloride complexes. It is suitable for cadmium separation from cobalt, copper, zinc, and manganese [28,29].

The present work aims to recycle and separate different valuable constituents of spent Ni–Cd batteries in a pure state using successive different separation techniques (solvent extraction for cadmium, adsorption for cobalt, and precipitation for nickel). Significant physical chemistry factors were investigated to develop the solvent extraction and adsorption approach, solvent, adsorbent concentration, pH, temperature, and extraction time.

## 2. Results and Discussion

### 2.1. Precipitation of Iron

The solution was treated with 1:1 of 32% hydrogen peroxide under agitation for 65 min at 60 °C. A green solution was produced at pH 3.0. The solution pH was increased to pH 4.0 using 10 M NaOH, then raised to pH 5.0 using 32% ammonium solution. After warming for 11 min at 73 °C, iron(III) hydroxide was the obtained product and was identified using EDX analysis [30].

### 2.2. Factors Controlling Adsorption of Cadmium

#### 2.2.1. Effect of pH

Using 25 mL battery liquor, 0.1 M Na_2_SO_4_ was added into deionized water as inert salt to minimize phase separation problems linked to low ionic strength. The samples were equalized at different pH values with 25 mL 0.02 M Adogen 464/kerosene while maintaining the same phase rate because the pH of the solution substantially affects extraction of the metal ions.

**Figure 1:**
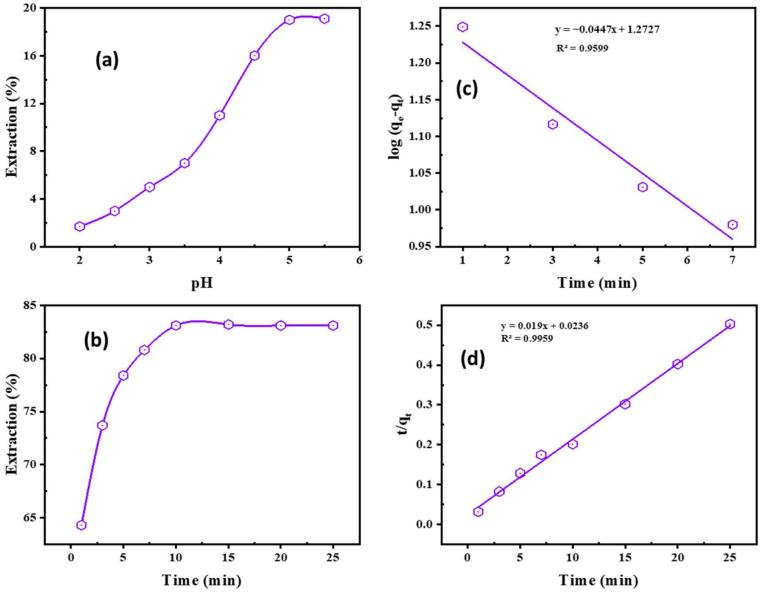
(**a**) Impacts of pH (0.1 M Na_2_SO_4_, 0.02 M Adogen 464, 5 min, O:A 1:1, 25 °C); (**b**) equilibration time on Cd^2+^ extraction (0.1 M Na_2_SO_4_, pH 5.0, 0.02 M Adogen 464, O:A 1:1, 25 °C); (**c**) The pseudo-1st-order; (**d**) pseudo-2nd-order modeling of Cd^2+^ extraction by Adogen 464.

The pH was in the range 2.0–5.5. The pH recorded after extraction is 5.0 which was greater than the original pH value. Subsequently, it declined with a rise in the original pH for the ionic fluids. As the pH of the solution increases, Cd^2+^ extraction increases; however, at pH 5, the percentage of cadmium extraction was insufficient, with just 19% extraction (Figure 1a). Increased pH with higher levels of Adogen 464 resulted in a higher percentage of cadmium extraction [31]. This may be because acid competes with Cd^2+^ ions or bisulfate ions at low pH values, hindering Cd^2+^ extraction. As the pH increased, extractable Cd^2+^ complexes were generated, which improved the extraction ratio.

#### 2.2.2. Effect of Time

The kinetics of Cd^2+^ removal was tested with the ionic liquid Adogen 464 in kerosene and with a 25 mL solution of leaching liquor and 0.1 M Na_2_SO_4_ in pH 5.0; the influence of balanced cadmium (II) removal time was investigated. As demonstrated in Figure 1b, the extraction efficiency improved gradually with the growth in contacting time until it reached its maximum efficiency at 10 min of contact time, which is sufficient to achieve equilibrium, after which the extraction efficiency became constant with any further increase in the contact time. 

The rate of cadmium ion extraction using Adogen 464 is described by its kinetics. The kinetic parameters assist in assessing sorption rates and offer necessary details for extraction approach setup and modelling. The Cd^2+^ extraction process by Adogen 464 and the rate constants were determined using pseudo-1st and 2nd-order modelling. The following mathematical Equation (1) describes the pseudo-1st-order modelling [32,33,34]:

(1) $$\log\left(q_{e}-q_{t}\right)=\log q_{e}-\left(\frac{K_{1}t}{2.303}\right)$$

The following formula describes the quantity of metal generated per unit weight (*q_e_*) and the quantity extracted per unit time (*q_t_*) increase: (*t*, min^−1^). As Figure 1c illustrates, the extraction rate of the 1st order follows a straight line when plotting log (*q_e_* − *q_t_*) vs. *t* values. To discover the practical data in Table 1, one can apply the pseudo-1st-order modelling, which matches the results shown in Table 1. A good correlation was established between the calculated value of *q_e_* at a modest extraction rate of (*K*_1_ = 0.1029 mg/g), which is around 18.74 mg/g. This is very different from the practical determining capability of 49.712 mg/g.

It is now apparent that the 1st-order modelling and the empirical data are not alike, and therefore the modelling is not suitable for the system under investigation. Conversely, the pseudo-2nd-order modelling is symbolized by the subsequent Equation (2) [35,36,37,38]:

(2) $$\frac{t}{q_{t}}=\frac{1}{K_{2}q_{e}^{2}}+\frac{t}{q_{e}}$$

The rate constant is given as *K*_2_ (g/mg·min). The slope of the linear plotting is 1/*q_e_*, and the intercept is 1/*q_e_*^2^. From the model in Figure 1d, and the data in Table 2, it is obvious that both the theoretical and experimental uptakes are close, and the correlation coefficient *R*^2^ is 0.9959 is higher than the 1st order one. The obtained data establish that the 2nd-order modelling is adapted to the extraction system.

#### 2.2.3. Effect of Adogen 464 Concentration

**Figure 2:**
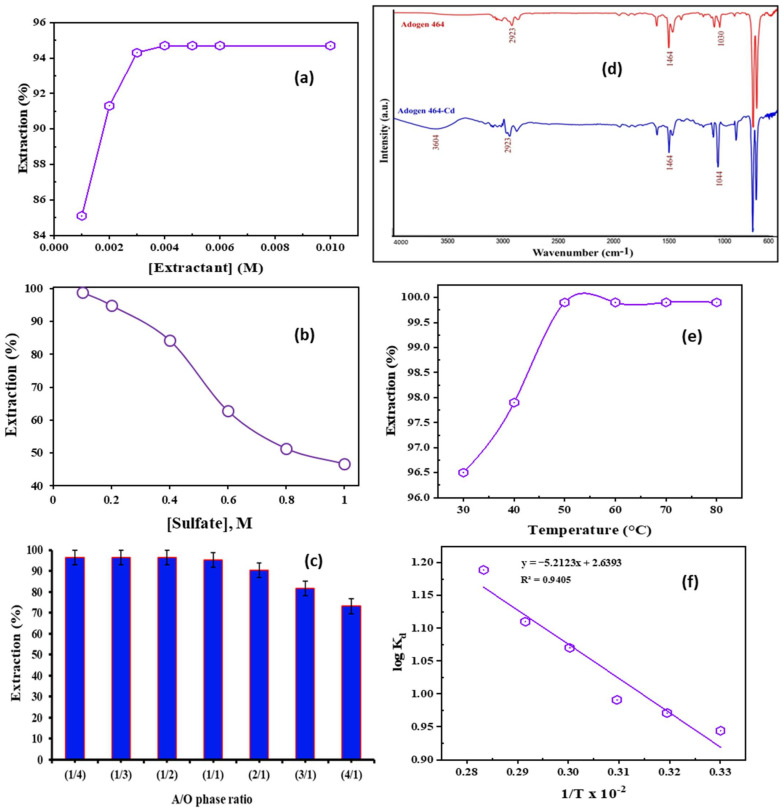
(**a**) Impact of Adogen 464 concentration upon Cd^2+^ extraction (0.1 M Na_2_SO_4_, pH 5.0, O:A 1:1, 25 °C, 10 min); (**b**) Impact of sulfate concentration upon Cd^2+^ extraction (0.2 M Adogen 464, pH 5.0, O:A 1:1, 10 min, 25 °C); (**c**) Impact of aqueous to organic ratio upon Cd^2+^ extraction (0.2 M Adogen 464, pH 5.0, 0.1 M Na_2_SO_4_, 10 min, 25 °C); (**d**) FTIR spectra of 0.2 M Adogen 464/toluene and cadmium/Adogen 464/toluene); (**e**) Impact of temp. upon Cd^2+^ extraction (0.2 M Adogen 464, pH 5.0, 0.1 M Na_2_SO_4_, O:A 1:1, 10 min); (**f**) Influence of temp. on Cd^2+^ partition coefficient (0.2 M Adogen 464, pH 5.0, 0.1 M Na_2_SO_4_, O:A 1:1, 10 min).

A total of 25 mL battery leach liquor, and extractant concentrations between 0.001 and 0.1 M, were used in the experiments to examine ionic liquid concentration impact on Cd^2+^ extraction. The results showed that ionic liquid concentration had no marked effect on Cd^2+^ extraction. Figure 2a illustrates that the percent extraction of cadmium rose as the extractant concentration was increased. 0.2 M Adogen 464 was used to extract 99.9% of the cadmium ions from the solution. At pH 5.0, and with other parameters constant, contact time at room temperature was 10 min.

#### 2.2.4. Effect of Sulfate Concentration

To explore the influence of Na_2_SO_4_ at concentrations of 0.1–1.0 M upon Cd^2+^ recovery, experiments were conducted at room temperature using 0.2 M Adogen 464. The experiment presented in Figure 2b showed that the percentage of cadmium extraction in the aqueous feed decreased with an enlargement in the concentration of sulfate ions because of the salting-out effect for Adogen 464/kerosene.

Nayl (2010) described the relationship between quaternary ammonium extractants, which were employed to extract metal ions, as only occurring with a solvation reaction mechanism. The extraction of the Cd^2+^ mechanism from SO_4_^2−^ media by R_3_R’N.SCN obeyed the addition mechanism, and the removed dominant species were (R_3_R’N.CdSO_4_.SCN) [39].

3Cd^2+^_(aq)_ + 3SO_4_^2−^_(aq)_ + 2H^+^ + 3(R_3_R’N.SCN)_(org)_ ↔ 3(R_3_R’N.CdSO_4_.SCN)_(org)_ + 2H^+^

where R = C_10_H_21_, C_9_H_19_, C_8_H_17_ and R’ = CH_3_ groups. The extraction (*K_ex_*) constant for Adogen 464 is gained from Equation (3).

(3) $$K_{ex}=\frac{{\left[(R_{3}R\text{′}N.{\mathrm{CdSO}}_{4}.\mathrm{SCN})\right]}_{\left(\mathrm{org}\right)}^{3}}{{\left[{\text{Cd}}^{2+}\right]}_{\left(\mathrm{aq}\right)}^{3}{\left[{\mathrm{SO}}_{4}^{2-}\right]}_{\left(\mathrm{aq}\right)}^{3}{\left[(R_{3}R\text{′}N.\mathrm{SCN})\right]}_{\left(\mathrm{org}\right)}^{3}}$$

#### 2.2.5. Effect of Organic to Aqueous Ratio

The ratio between aqueous/organic phases was studied with various ratios or relations of aqueous leach liquid with the fitting organic solvent, generating the balance curve. The influence of varying the aqueous to organic phase ratio (A:O) upon Cd^2+^ extraction was examined using different aqueous/organic phase ratios from 4:1 to 1:4 at 25 °C, 10 min of shaking time, pH 5.0, and 0.2 M Adogen 464/kerosene. According to Figure 2c, the A:O ratio slightly influences the extraction efficiency in the case of increasing the organic volume. In the other case, however, the extraction efficiency decreases gradually with increasing the aqueous phase. Hence, the O:A ratio of 1:1 was selected as the optimal ratio, with 96.4% extraction.

Fourier infrared spectroscopy technique (FTIR) has been employed to detect many characteristic vibrational bands [40]; it can detect the functional groups of Adogen 464 before and after the extraction of cadmium. A study was conducted using Adogen 464, diluted in kerosene and presented in Figure 2d. The peak at 1030 cm^−1^ for Adogen 464 was assigned for –C–N stretching vibration that was transformed to 1044 cm^−1^ for Cd-Adogen 464 complex, after the bonding among Cd^2+^ and Adogen 464 happened. The wide absorption band at 3604 cm^−1^ for Cd-Adogen 464 was assigned for the stretching vibration of –OH of soluble moisture [41]. The distinctive peak of quaternary amine at 1464 cm^−1^ appeared in both spectra and was induced via (CH_3_)N^+^. The asymmetric –CH peaks (2923 cm^−1^) for a combination of Adogen 464 prior to and after extraction investigations were equivalent. 

#### 2.2.6. Effect of Temperature

The extraction rate may rise or decrease depending on the temperature of the environment. Temperatures ranging from 30 °C to 80 °C were used to scrutinize the temperature influence upon Cd^2+^ extraction via ionic liquid Adogen 464. The aqueous solution used in this experiment was the battery leach liquor with 0.1 M Na_2_SO_4_, pH 5.0, and 0.2 M Adogen 464 in kerosene, as shown in Figure 2e. Altogether, the outcomes demonstrated that temperature had a favourable influence on Cd^2+^ extraction. The extraction of Cd^2+^ ions rose as the temperature increased; it reached its maximum *E*% (99.9%) at 50 °C. From the successive van’t Hoff Equation (4) [42,43,44,45].

(4) $$\log K_{d}=\frac{-\Delta H^{{}^{\circ}}}{2.303RT}+\frac{\Delta S^{{}^{\circ}}}{2.303R}$$

The enthalpy (Δ*H*°) and entropy (Δ*S*°) were determined via the plot of log *K_d_* vs. 1/*T* (Figure 2f). From the relation of Δ*G*° = Δ*H*° − *T*Δ*S*°, the values of Δ*G*° were computed and are listed in Table 2.

The positive significance value of the ∆*H*° in Table 2 shows that the extraction of cadmium ions through Adogen 464 is an endothermic mechanism, which is confirmed by the temperature impact that affects the *E*% of Cd^2+^ ions. Negative ∆*G*° values show that the suggested extraction contrivance is spontaneous and feasible. The Arrhenius relation is important as the straight-line slope in Figure 2f is applicable for assessing the apparent activation energy (*E_a_*) of cadmium ions extraction at different temperatures [46]. Values are calculated from the following Equation (5) [47].

(5) $$\log K_{d}=\frac{-2.303E_{a}}{RT}+\log A$$

The partition coefficient, *K_d_*, and *E_a_* (kJ·mol^−1^) denote the extraction activation energy, *R* (8.314 J·mol^−1^·K^−1^ is the molar gas constant), and *T* is the absolute temperature in Kelvin. Cadmium ions extraction required 1.882 kJ·mol^−1^ of activation energy, and the Arrhenius constant is 436.4. This signifies that Cd^2+^ ions extraction by Adogen 464 is an endothermic approach, which means that the reaction requires energy to be completed.

### 2.3. Cadmium Stripping from Cadmium/Adogen 464 and Preciptation

**Figure 3:**
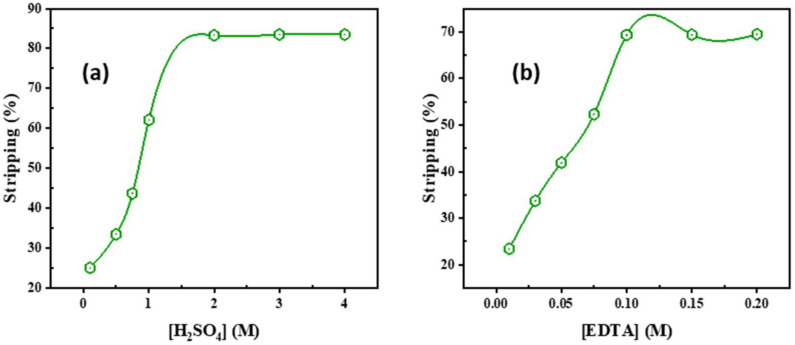
Influence of (**a**) H_2_SO_4_; (**b**) EDTA concentration upon Cd^2+^ stripping from the loaded Adogen 464.

The removal of Cd^2+^ ions from the loaded Adogen 464 in toluene was accomplished using different concentrations of H_2_SO_4_ and EDTA. Figure 3a demonstrates that raising the acid concentration from 0.1–2.0 M causes an increase in Cd^2+^ stripping from 25% to 83.5%. The use of 2.0 M H_2_SO_4_ and/or 0.1 M EDTA in two successive stages at an O:A ratio of 1:2 resulted in a significant increase in Cd^2+^ stripping from 23.4% to 69.5% with an increase in EDTA concentration from 0.01 to 0.1 M, resulting in the complete removal of the entire cadmium content of the solvent, as shown in Figure 3b. As a result, 2.0 M H_2_SO_4_ was selected as the most effective stripping agent for this work.

**Figure 4:**
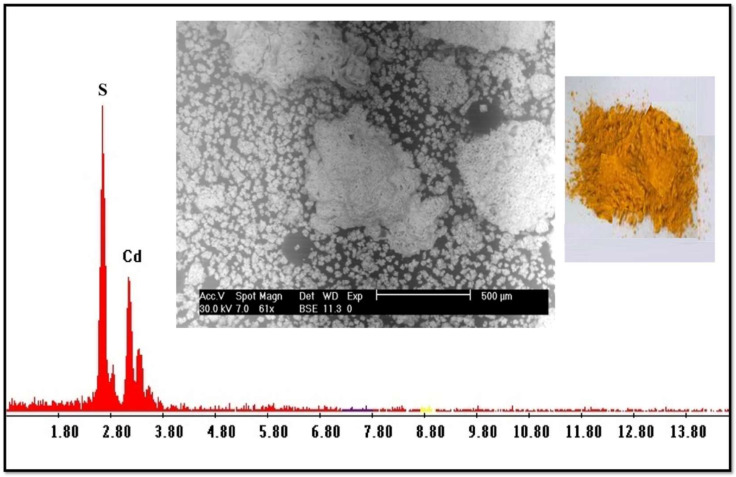
SEM-EDX chart for CdS product.

The sulfate solution containing Cd^2+^ produced from the stripping process was subjected to precipitation using 0.5% Na_2_S solution. Adjusting the pH to 1.25 at room temperature, a yellow precipitate of CdS was formed; it was separated via filtration and dried in the oven at 70 °C. The obtained product was about 2.0 g. The product has also been subjected to SEM-EDX analysis, as shown in Figure 4, which reveals a maximum purity.

### 2.4. Adsorption of Cobalt Using Prepared Silica Adsorbant

The leachate is now free of cadmium and iron and contains nickel and cobalt. The remaining solution was subjected to the selective separation of Co^2+^ from Ni^2+^ utilizing a new adsorbent, PTU-MSA.

#### 2.4.1. Characterization of PTU-MSA

**Figure 5:**
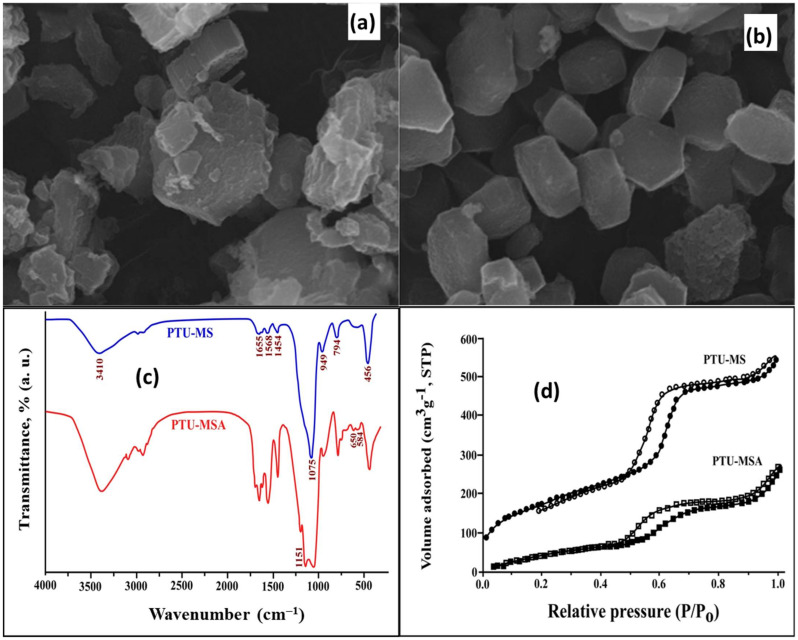
(**a**) SEM photograph after sulfonation of PTU-MS; (**b**) SEM photograph after sulfonation of PTU-MSA; (**c**) FT-IR spectra of PTU-MS and PTU-MSA materials; (**d**) N_2_ adsorption-desorption isotherm curves of PTU-MS and PTU-MSA.

The morphology of PTU-MS was gained via an electron scanning microscope (SEM-EDX). The matrix mainly consisted of a hexagonal formation and showed a rather homogeneous particle size of about 0.85 mm PTU-MSA adsorbent (mean size) [48]. It was different from PTU-MS (Figure 5a,b). Moreover, the PTU-MS was analyzed using FTIR. The data showed major typical peaks in the range of 2900–3000 cm^−1^ due to aliphatic stretching of C–H. The feature at 1655 cm^−1^ is C=O for urea, that at 3410 cm^−1^ is due to phenolic –OH, and peaks at 1454 cm^−1^ and 1568 cm^−1^ correspond to N–H and N–C, respectively. Three bands are attributed to silica at 1075, 794, and 456 cm^−1^ of Si–O–Si. Moreover, a peak at 949 cm^−1^ is assigned to Si–OH [49,50,51,52,53,54].

The spectrum of sulphonic acid-modified PTU-MSA shows new bands at 1151 cm^−1^ and a specific peak is recorded at around 584 cm^−1^, resulting from the SO_3_ group. The peak at 650 cm^−1^ is dispersed due to the stretching vibration of the S–N. The S–N band was predicted to overlap with the Si–O stretching band of the identical area of energy (Figure 5c). The spectrum of FT-IR confirmed that the surface of prepared PTU-MS and PTU-MSA material was successfully functionalized and synthesized [55,56].

The nitrogen adsorption/desorption isotherm curves (BET) of the two samples (PTU-MS and PTU-MSA) were obtained through steep condensing/evaporation capillary stages [57,58,59]. Recognizable H1 hysteresis loops were shown for the adsorption/desorption of nitrogen in the two samples (PTU-MS and PTU-MSA), which are typical for mesoporous materials with cylindrical mesoporous following the IUPAC classification. The PTU-MSA’s specific surface area is decreased due to the sulphonic acid group’s incorporation into the PTU-MS framework. The results in Table 3 along with Figure 5d, showed a reduction in the pore diameter and pore volume indicating successful incorporation of the group.

#### 2.4.2. Factors Controlling the Adsorption Process of Cobalt

The adsorption of cobalt was initially tested using PTU-MSA from a synthetic solution. It was then applied on the leach liquor free of cadmium and iron, containing only cobalt and nickel.

1. Impact of pH

**Figure 6:**
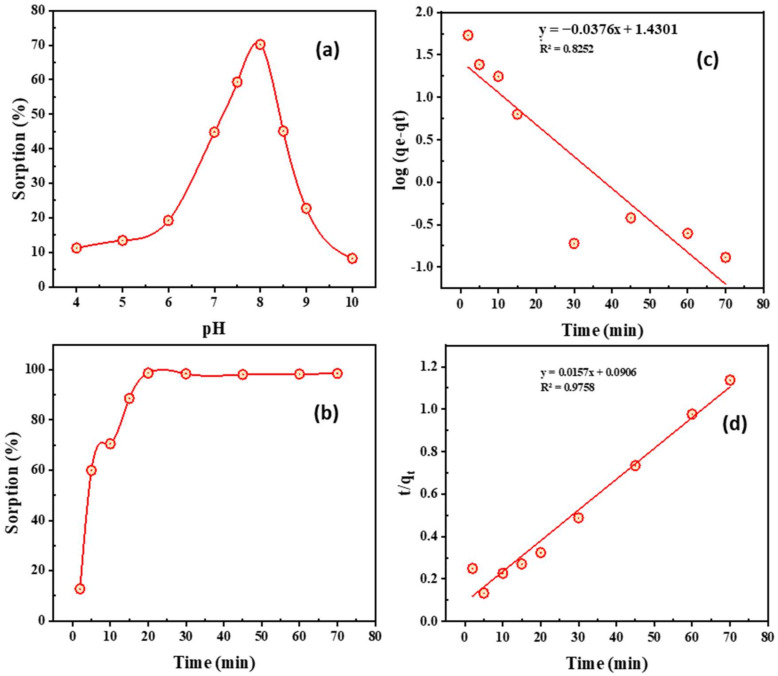
(**a**) impact of pH upon Co^2+^ adsorption (20 mL of 250 mg/L Co^2+^, 10 min, 0.1 g of PTU-MSA, 25 °C); (**b**) Impact of contacting time upon Co^2+^ adsorption (20 mL of 250 mg/L Co^2+^, pH 8.0, 0.08 g of PTU-MSA, 25 °C); (**c**) 1st-order kinetic plot; (**d**) 2nd-order kinetic plot of PTU-MSA’s Co^2+^ adsorption (20 mL of 250 mg/L Co^2+^, pH 8.0, 0.08 g of PTU-MSA, 25 °C).

The pH has a vital role in reducing or increasing the adsorption selectivity of efficiently hydrolysable metal ions. As already established, most metal ions are hydrated in water [60]. Figure 6a indicates the pH dependency of Co^2+^ ions on PTU-MSA. The Co^2+^ ions form insoluble aqueous complexes with increased pH, when Co^2+^ ions undergo hydrolysis reactions in water.

The solution pH influences the speed of surface reactions. The adsorption capacity changes with pH scope mostly due to the impact of pH upon the adsorption aspects of the PTU-MSA surface. Over the pH range 4.0–10.0, Co^2+^ adsorption efficiency increased with higher pH, as demonstrated in Figure 6a. The adsorption conditions were kept constant at Co^2+^ concentration of 250 mg/L, 10 min, and 0.1 g of PTU-MSA at room temperature, while the pH was varied.

The results shown in Figure 6a indicate that the Co^2+^ adsorption on the surface of PTU-MSA steadily rose with the solution pH rising to the highest or maximal at pH 8. The Co^2+^ species exclusively exist in a divalent ionic state at this pH, enhancing the removal of the adsorbent from the solution. The pH effect can be explained by the presence in working solutions with pH values just below 8.0 of various ionic shapes such as Co^2+^, Co(OH)^+^, Co(OH)_2,_ and Co(OH)_3_^−^, which reduce the overall effectiveness of the Co^2+^ removal [61,62]. PTU-MSA has the maximum adsorption efficiency at pH 8.0. At rising pH levels (pH > 8.0), the Co^2+^ was precipitated as cobalt hydroxide. This outcome displays the pH effect on the adsorbent.

2. Impact of time and kinetics

The foremost important feature of cobalt adsorption was the adsorption time. Sequences of experiments with 0.08 g PTU-MSA adsorbent were examined at differing contact times (5–70 min) while the rest of the experimental conditions were maintained constant. As shown in Figure 6b, Co^2+^ sorption capacity rose by 5 min to 61.69 mg·g^−1^ upon PTU-MSA. However, the Co^2+^ ions uptake was not affected when the contact period extended after 20 min. As a result, the balance was extended to 20 min, and the adsorption conditions of cobalt were then improved.

The kinetics of the adsorption process of Co^2+^ regulates the Co^2+^ uptake rate and, in turn, the adsorbate residence time was controlled by this rate at the solid-solution interface. Lagergren’s 1st- and 2nd-order kinetic modellings derived PTU-MSA rate constants were used to quantify Co^2+^ adsorption [63,64]. The correlation coefficient was utilized to measure the consistency of the investigational outcomes by the values estimated through the two models (*R*^2^). A higher *R*^2^ assessment suggests that Co^2+^ adsorption kinetics were accurately characterized by a particular model.

The pseudo-1st- and pseudo-2nd-order kinetics (see Equations (1) and (2)) were shown in Table 4 and Figure 6c,d. The 2nd-order correlation coefficient is 0.9758 and the theoretical uptake is 61.69 mg·g^−1^, which resembles the investigational finding. The result suggests that Co^2+^ adsorption upon PTU-MSA hinges on the initial Co^2+^ concentration. Thus, pseudo-2nd-order kinetics anticipates the adsorption performance throughout the extent of the whole concentration examined [61].

3. Impact of PTU-MSA dose

**Figure 7:**
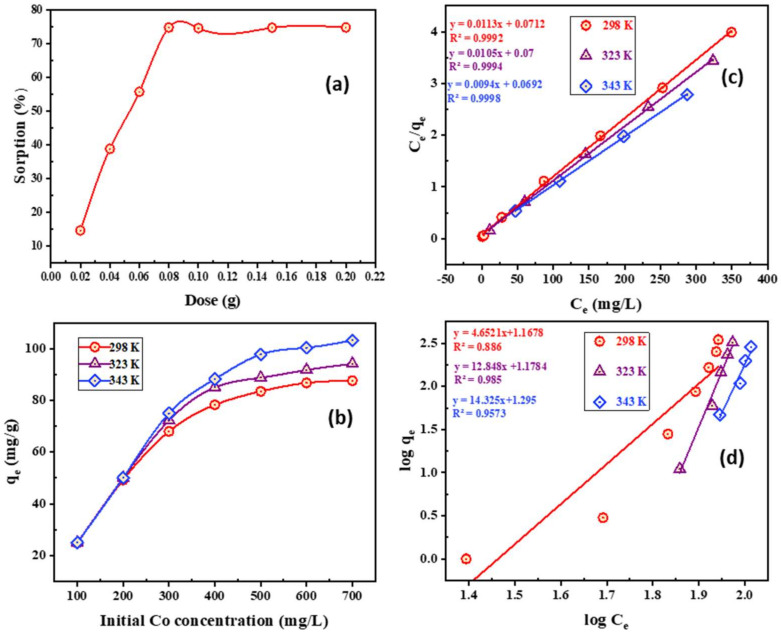
(**a**) Impact of PTU-MSA dose upon Co^2+^ adsorption (20 mL of 250 mg/L Co^2+^, pH 8.0, 20 min, 25 °C); (**b**) Impact of initial Co^2+^ concentration upon PTU-MSA uptake (20 mL of 250 mg/L Co^2+^, pH 8.0, 20 min, 0.08 g PTU-MAS); (**c**) Langmuir isotherm model; (**d**) Freundlich isotherm mode for Co^2+^ adsorption upon PTU-MSA (20 mL of 250 mg/L Co^2+^, pH 8.0, 20 min, 0.08 g PTU-MAS).

The influence of PTU-MSA dosage upon Co^2+^ adsorption was investigated over the range 0.02–0.2 g. As demonstrated in Figure 7a, with augmented PTU-MSA dosage from 0.02 to 0.08 g, the Co^2+^ uptake improved [58,59]. The Co^2+^ adsorption upon PTU-MSA does not differ substantially when the PTU-MSA dosage is more than 0.08 g with the same constant Co^2+^ concentration in the medium. For the subsequent experiments, 0.08 g of PTU-MSA was therefore chosen.

4. Impact of initial Co^2+^ concentration and adsorption isotherms features

For three constant temperatures (298, 323, and 343 K), the effect of the initial Co^2+^ concentration was explored. The Co^2+^ adsorption ability improved as the initial Co^2+^ concentration was increased, according to the results reported in Figure 7b. Increased Co^2+^ ion concentrations enhanced the forces in the aqueous and solid phase that suppress the mass transfer. In addition, a beneficial effect was seen on the uptake of Co^2+^ ions in PTU-MSA which shows the process could have an endothermal origin.

Two isothermal models have been utilized to explore the interaction and distribution mechanism between Co^2+^ and solid interface, the isotherms of Freundlich and Langmuir. The Langmuir isothermal pattern implies that monolayers with homogenous binding sites form on the surface of the PTU-MSA (Figure 7c) [65,66,67]. The model is usually described by Equation (6):

(6) $$\frac{C_{e}}{q_{e}}=\frac{1}{bq_{max}}+\frac{C_{e}}{q_{max}}$$

where *C_e_* (mg·L^−1^) is the equilibrium concentration, *q_e_* (mg·g^−1^) is the equilibrating uptake of adsorbed Co^2+^, *q_max_* (mg·g^−1^) is maximal sorption capability, and *b* (L·mg^−1^) is equilibrating adsorbance constant. Freundlich modeling is presumed such that adsorption performance is carried out with variable binding energies in scenarios with a heterogeneous surface (Figure 7d) [68,69,70,71,72,73]. The Freundlich modeling is expressed as Equation (7):

(7) $$\ln q_{e}=\ln k_{f}+\frac{1}{n}\ln C_{e}$$

where *k_f_* (mg·g^−1^) is the uptake of the Freundlich continuum and *n* is the sorption intensity parameter. Table 5 shows that the result agrees more with the Langmuir than Freundlich isothermal for Co^2+^ adsorption. This may be due to the greater correlation coefficient (*R*^2^ = 0.9992) and the maximal sorption capacity at all three temperatures, which are closer to the experimental. The outcomes showed that there was sorption upon a homogenous monolayer surface and that the energy of every metal-binding place is the same [74,75,76]. In the Freundlich isotherm, the computed value is 1/*n* higher than 1.0 (Table 5), which showed normal adsorption. It may be inferred that the higher the *k_f_*, the higher is the adsorption intensity [77,78].

When the values of the separation factor constant (*R_L_*) are calculated, the degree of adsorptive capacity of PTU-MSA towards Co^2+^ can be predicted, and this gives an indicator of whether the adsorption development can occur. A suitable environment for Co/PTU-MSA adsorption exists when the *R_L_* values fall within the range of 0 to 1.0. It is found that the relative lightness (*R_L_*) ranges between 0.003614 and 0.10626, indicating that the PTU-MSA is capable of adsorbing Co^2+^ from the aqueous phase. The following thermodynamic evaluation factors can prove this Equation (8):

(8) $$R_{L}=\frac{1}{1+K_{L}C_{o}}$$

5. Thermodynamic parameters for systems of cobalt adsorption

The intercept and slope of the plot of log *K_d_* vs. 1/*T* for the cobalt adsorption system were used to compute the values of Δ*H*° and Δ*S*°, besides the values of Δ*G*° from Equation (4) [79]. The thermodynamic parameters are shown in Table 6. Both Δ*H*° and Δ*S*° are positive, and *T*Δ*S*° is larger than Δ*H*°.

These findings support the idea that this adsorption mechanism is endothermic. Positive values of Δ*S*° indicate that there is a strong affinity between PTU-MSA and Co^2+^ ions. Furthermore, a decline in negative Δ*G*° quantities with increasing temperature demonstrates that sorption processes are more effective at more elevated temperatures, most likely because ions are more mobile in the solution [46].

6. Impact of Ni^2+^ ion concentration on PTU-MSA selectivity for Co^2+^ adsorption

**Figure 8:**
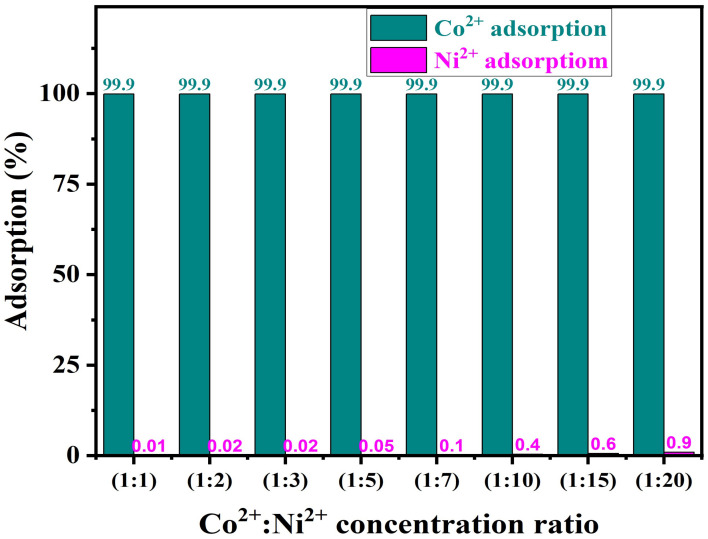
Influence of nickel concentration upon the selectivity of PTU-MSA for Co^2+^ ions.

In this experiment, we use a mixture of Co^2+^ and Ni^2+^ in the same solution, while changing the concentration of Ni^2+^ to study the influence of Ni^2+^ on the selectivity of PTU-MSA for Co^2+^ adsorption. The data in Figure 8 demonstrate that increasing the concentration of nickel to more than 15-fold the concentration of Co^2+^ ions has no influence on the adsorption of cobalt. There is no affinity of PTU-MSA for adsorption of Ni^2+^ ions. This study covered the application of PTU-MSA to leach liquor that contained only nickel and cobalt ions.

### 2.5. Desorption and Precipitation of Cobalt

**Figure 9:**
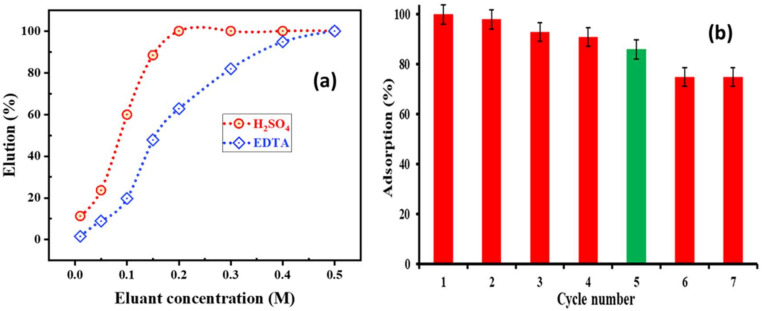
(**a**) Effect of eluents concentration on elution of cobalt loaded on PTU-MSA; (**b**) Reusability of PTU-MSA.

The cobalt was eluted from Co/PTU-MSA to a varied phase ratio in the range of 0.01–0.5 M for a 5 min duration using different concentrations of sulfuric acid and ethylene diamine tetraacetic acid (EDTA). Metal values were examined in the aqueous phase. The elution percentage of Co^2+^ vs. H_2_SO_4_ concentration (M) is shown in Figure 9a. The cobalt desorption grew from 10.8 to 99.9% by enhancing the concentration of H_2_SO_4_ from 0.01 to 0.2 M. In case of EDTA, the maximal desorption of Co^2+^ was realized at 0.5 M; the highest elution percentage in H_2_SO_4_ concentration, 0.3 M H_2_SO_4_ was unique as the greatest eluting agent.

At this juncture, to identify how often the adsorbent can be used for the adsorption process, and whether its effectiveness changes or not, the regeneration process has been carried out seven times after every adsorption cycle. Figure 9b shows that the adsorption capacity was reduced with higher numbers of cycles. In the first 4 cycles, the positive results were evident. However, the adsorption percentage fell in cycle number 5 compared to the first cycle from around 99 to 84%. Cycles 6 and 7 were extremely close in their adsorption percentages. A decrease in adsorption percentage may be due to poisoning of the active sites or partial leaching.

**Figure 10:**
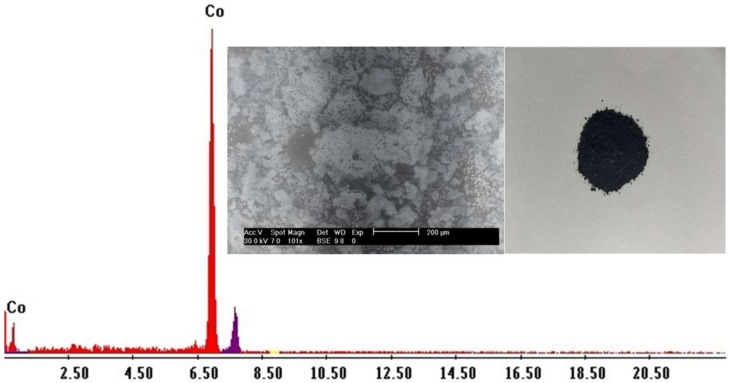
EDX and SEM analysis of CoO.

After desorption of cobalt ions, the precipitation of Co^2+^ was achieved by increasing the pH of the solution pH to 9.0 using NH_4_OH; a pale pink precipitate of Co(OH)_2_ was formed. It was filtrated and cleaned with distilled H_2_O numerous times, after which the precipitate was dried at 60 °C for 2 h. Later, it was calcined at 900 °C to obtain a cobalt (II) oxide (CoO) product. The final product was analyzed by SEM-EDX (Figure 10), which showed that the product is entirely cobalt.

### 2.6. Nickel Recovery

**Figure 11:**
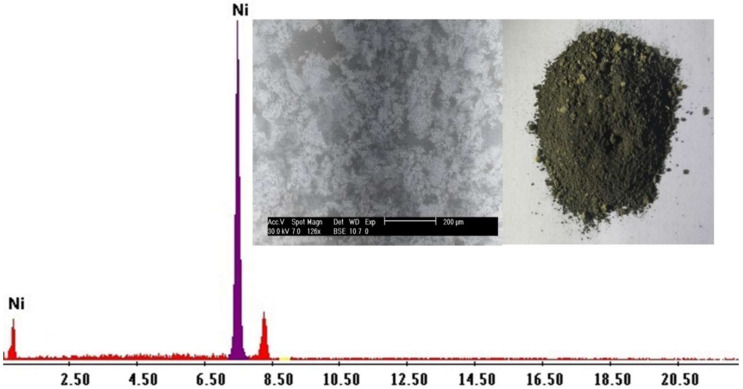
EDX and SEM analysis of NiO.

The final solution contains only nickel ions after separation of cadmium and cobalt from the Cd–Ni batteries leach liquor. 200 mL of the sulfate leach liquor contains about 0.823 g of Ni. This has been precipitated at pH 8.25 using 10% NaOH, added to the Ni solution at room temperature. After filtration, the precipitate Ni(OH)_2_ was washed several times with distilled H_2_O to eliminate any impurities and ignited at 650 °C for 1.5 h. The NiO product was recovered and identified using chemical and SEM-EDX techniques, as shown in Figure 11. The recovery of Ni was attained 98% with a purity ≥ 97%. In fact, the interference problem from Co^2+^ ions has been overcome by the prior removal of Co^2+^ ions.

## 3. Materials and Methods

### 3.1. Chemicals and Instruments

**Figure 12:**
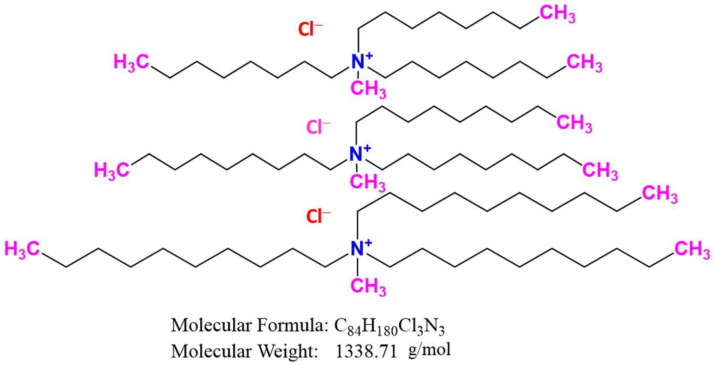
Structure of Adogen^®^ 464.

Cadmium sulfate, xylenol orange, Adogen 464, KSCN, and sulfuric acid were supplied from Riedel-Dehaen AG and Merck, Merck, Darmstadt, Germany. 3-(Triethoxysilyl) propylisocyanate (95%), poly 2,4-diaminophenol (98%), and ethylene glycol were bought from Sigma-Aldrich chemicals, St. Louis, MO, USA. Tetraethyl orthosilicate, nickel nitrate, block polypropylene glycol-block polyethylene glycol (P123, EO20PO70EO20, MW = 5800 g/mol), chromium nitrate, cobalt nitrate anhydrous, chlorosulphonic acid, triethylamine trihydrochloride and copper nitrate anhydrous were obtained from Merck, Darmstadt, Germany. Distilled water was utilized in all processes. The structure and molecular weight of Adogen 464 are shown in Figure 12. A pH meter (systronics μ pH-System 362) was used to monitor pH for the aqueous phase before and after extraction. An ICP-OES spectrometer (OPTIMA 5300 DV, PerkinElmer, Waltham, MA, USA) was utilized for elemental analysis. A mass balance provided the metal quantities extracted by the solvent extractor. FTIR Affinity-1S (Shimadzu, Kyoto, Japan) was applied to detect the FTIR spectra of samples.

### 3.2. Preparation of Cd–Ni Batteries Leachate

**Figure 13:**
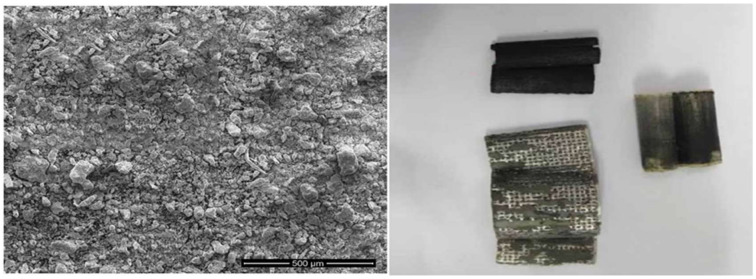
SEM image of Ni–Cd battery powder and a digital photograph of a dismantled spent Ni–Cd battery.

The spent batteries were manually broken into various fractions. Plastic and paper wastes were removed while the electrodes (anode and cathode-active materials) were separated and mixed, ground then washed with distilled water to remove electrolyte (KOH). Figure 13 shows the SEM diagram powder of a deconstructed Ni–Cd battery. After that, the mixture was roasted at 550 °C to oxidize metallic cadmium and decompose cadmium and nickel salts. The net weight of the mixture was about 50 g; the leaching process occurred using 20% H_2_SO_4_, solid/liquid (S/L) of 1/5 at 80 °C for 6 h. Before leaching, a combination of conventional and tooling methods was utilized to scrutinize the chemical formatting of the examined powder (originated from Ni–Cd battery) [80]. Table 7 shows the concentrations of the components that agree with published data before and after the leaching procedure [81,82,83]. Therefore, the solution contained some iron, which had to be precipitated. The iron-free leaching solution was also employed for selective extraction of Cd^2+^ ions using Adogen 464.

### 3.3. Preparation of Adogen^®^ 464 for Extraction Process

Adogen^®^ 464 (30%) was converted from chloride to thiocyanate form; this was exposed to 1.5 M KSCN solution, at a 1:1 ratio of water to organic material. It was agitated for 20 min. After two injections of organic solution with a new SCN^−^ solution, there were still traces of thiocyanate in the organic solution. After being in contact with the organic solution, thiocyanate titration was indicated by AgNO_3_ concentration [29]. An appropriate volume of Adogen^®^ 464 with commercial-grade kerosene (diluent) was diluted and/or dissolved to generate organic solutions of varied levels unless otherwise indicated. Concentration levels of the ionic fluid were determined.

### 3.4. Extraction Processes and Measurements

A 100 mL separation funnel with the aqueous phase of Cd^2+^ was balanced with organic Adogen 464 solvent at room temperature (except for temperature variation). After the water layer separation, the pH was determined with a pH meter. Each trial was explored three times, with error bars added on each occasion. Following extraction, the amount of a metal in water increases when it moves from the aqueous stage to the organic stage, according to the equation (Equation (8)). [*M*]*_o_* equals the metal amount in the organic layer, and [*M*]*_A_* equals the metal amount in the aqueous layer following extraction:

(9) $$D=\frac{{\left[M\right]}_{o}}{{\left[M\right]}_{A}}=\frac{{\left[M\right]}_{i,A}-{\left[M\right]}_{A}}{{\left[M\right]}_{A}}$$

(10) $$D=\frac{{\left[M\right]}_{i,A}-{\left[M\right]}_{A}}{{\left[M\right]}_{A}}\times \frac{V_{A}}{V_{o}}$$

*V_A_* and *V_O_* are volumes of aqueous and organic layers. Hence, the extraction efficiency was applied as observed [84]:

(11) $$E\%=\frac{100D}{D+\left(\frac{V_{A}}{V_{o}}\right)}$$

### 3.5. Preparation of Precursor, 1,1′-(4-hydroxy-1,3-phenylene)bis(3-(3-(triethoxysilyl)propyl)urea (PTU)

**Figure 14:**
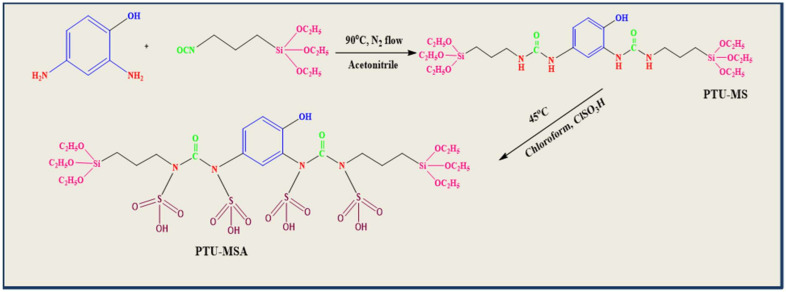
Preparation of PTU-MS and PTU-MSA.

A 2,4-Isocyanate of propyl-di-aminophenol (1.2 g) and 3-(triethoxysilly)propyl isocyanate (5.0 g) was liquefied at the concentration of 1:2 in 85 mL dry acetonitrile (Figure 14). The mix was warmed to 85 °C for 24 h via static reflux. Thin-layer chromatography (TLC) was utilized to assess reaction advancement. The solvent was then evaporated to produce a white precipitate during over 5 h in dry hexane. The puffed white outcome was purified, cleaned with hexane, and dried with a vacuum.

### 3.6. Synthesis of PTU Bridged Mesoporous Organosilica (PTU-MS)

N1,1′-(4-hydroxy-1,3-phenylene) bis(3-(3-(triethoxysilyl)propyl)urea, a bridging mesoporous material, was prepared [85]. TEOS as the parent source of silica and the structure-directing agent, PTU as the organic bridging groups, and pluronic P-123 as a sample were used and mixed in a flask. The synthesized materials were designated PTU-MS. Solutions of P123, HCl, and water were mixed with a strong stirring at 35 °C in a typical process. The combination of 2 mixtures was allowed to mix for 24 h at the above temperature until white precipitation was established, and the heterogeneous blend aged with mixing for a further 24 h and 80 °C. The white precipitate was filtered, cleaned with H_2_O, and dried in air for various times.

Before using the produced adsorbent PTU-MS, it was subjected to washing with HCl (3 mL)–ethanol (150 mL) solution for 12 h at 25 °C to get rid of the surfactant template. This was performed three times until the surfactants had been eliminated. To obtain the final product, PTU-MS was filtered, cleaned with ethanol, and desiccated overnight at 60 °C.

Finally, 2.0 g of PTU-MS was dipped in chloroform, stirred at 45 °C for 1 h and mixed with 0.08 mL of chlorosulphonic acid (ClSO_3_H), to generate enough triethylamine to eliminate the released hydrochloric acid with 18 h of stirring (Figure 14). The yield was filtered, cleaned with excess chloroform, and vacuum-dried to gain organic-inorganic mesoporous silica improved by a group of sulphonic acids. The end product received the symbol PTU-MSA. The preparation of PTU-MS and PTU-MSA are illustrated in Figure 14.

## 4. Conclusions

The Scarp Ni–Cd batteries (50 g) were treated with 20% H_2_SO_4_, with the 1:5 solid/liquid (S:L) ratio at 80 °C for 6 h. The leaching efficiency of Cd, Fe, and Co was nearly 100%, whereas the leaching efficiency of Ni was 95%. Both newly prepared materials were confirmed via FTIR and SEM techniques. Adogen 464 was used for Cd^2+^ extraction, followed by precipitation as a yellow CdS product with 0.5% Na_2_S solution by setting the pH at 1.25, at room temperature. PTU-MS silica adsorbent was used as an ion exchanger for Co^2+^ ions. 0.3 M H_2_SO_4_ was employed as an eluant and the precipitation of cobalt was achieved at up to pH 9.0 as Co(OH)_2_. The kinetic and thermodynamic parameters were also investigated. Lastly, nickel was directly precipitated at pH 8.25, using a 10% NaOH solution at ambient temperature.

## Figures and Tables

**Table 1:** The parameters of Cd^2+^ extraction kinetic by Adogen 464.

Pseudo-1st Order			Exp. Capacity *q_e_*, mg/g	Pseudo-2nd Order		
*q_e_*	*K*_1_	*R* ^2^	49.712	*q_e_*	*K*_2_	*R* ^2^
18.737	0.1029	0.9599		52.632	0.0153	0.9959

**Table 2:** Thermodynamic parameters acquired from Cd^2+^ extraction temperature investigation.

Δ*H*° (kJ·mol^−1^)	Δ*S*° (J·mol^−1^·K^−1^)	Δ*G*° (kJ·mol^−1^)					
9.981	0.0505	303 K	313 K	323 K	333 K	343 K	353 K
		−15.302	−15.808	−16.313	−16.818	−17.323	−17.829

**Table 3:** Physico-chemical properties of PTU-MS material sorption, for the examination of N_2_ prior and after incorporation of a sulphonic group.

Materials	Specific Surface Area (m^2^·g^−1^)	Pore Volume (cm^3^·g^−1^)	Pore Diameter (Å)
PTU-MS	634	0.71	68
PTU-MSA	357	0.42	55

**Table 4:** Kinetic factors of Co^2+^ adsorption upon PTU-MSA.

Pseudo-1st Order			Exp. Capacity *q_e_* (mg·g^−1^)	Pseudo-2nd Order		
*q_e_*	*K*_1_	*R* ^2^	61.69	*q_e_*	*K*_2_	*R* ^2^
26.922	0.08659	0.8252		63.692	0.00272	0.9758

**Table 5:** Langmuir and Freundlich data for Co^2+^ adsorption by PTU-MSA.

Temp °C	Langmuir Isotherm			*q_e_* (mg·g^−1^)	Freundlich Isotherm		
	*b*	*q_max_*	*R* ^2^		*k_f_*	1*/n*	*R* ^2^
25	14.045	88.496	0.9992	87.625	14.716	4.6521	0.886
50	14.288	95.24	0.9994	94.125	15.079	12.848	0.985
70	14.451	106.38	0.9998	103.2	19.724	14.325	0.9573

**Table 6:** Factors controlling the thermodynamics of Co^2+^ adsorption upon PTU-MSA at different temperatures.

Δ*H*° (kJ·mol^−1^)	Δ*S*° (J·mol^−1^·K^−1^)	Δ*G*° (kJ·mol^−1^)		
15.921	7.018	298 K	323 K	343 K
		−2.075	−2.251	−2.391

**Table 7:** Chemical composition of consumed Ni-Cd battery powder.

Elements	Chemical Configuration of Powder Ni–Cd Batteries (g/kg)	Elements Concentration in Ni–Cd Batteries Leachate (g/L)	Leaching Efficiency, %
Ni	21.89	20.80	94.90
Cd	13.31	13.31	99.90
Co	0.85	0.85	99.90
Fe	2.71	2.71	99.90

## Data Availability

Not applicable.
